# Plasma Translocator Protein Levels and Outcomes of Acute Ischemic Stroke: A Pilot Study

**DOI:** 10.1155/2018/9831079

**Published:** 2018-06-21

**Authors:** Wen-Hung Chen, Hsu-Ling Yeh, Chiung-Wen Tsao, Li-Ming Lien, Arthur Chiwaya, Javad Alizargar, Chyi-Huey Bai

**Affiliations:** ^1^Department of Neurology, Shin Kong Wu Ho-Su Memorial Hospital, Taipei, Taiwan; ^2^Department of Nursing, Chung Hwa University of Medical Technology, Tainan, Taiwan; ^3^School of Medicine, College of Medicine, Taipei Medical University, Taipei, Taiwan; ^4^School of Public Health, College of Public Health, Taipei Medical University, Taipei, Taiwan

## Abstract

Translocator protein 18 kDa (TSPO) has been used as a biomarker of brain injury and inflammation in various neurological diseases. In this study, we measured the level of TSPO in acute ischemic stroke patients and determined its association with the degree of stroke severity and its ability to predict stroke functional outcomes. In total, 38 patients with moderate to severe acute ischemic stroke were enrolled. Demographic information, cerebral risk factors, and stroke severity were examined at the baseline. The National Institutes of Health Stroke Scale, modified Rankin Scale, and Barthal Index were assessed at discharge as measures of poor functional outcomes and severe disability. The baseline fasting plasma TSPO level was assessed within 24 h after the incident stroke and during hospitalization (on days 8–10). The proportion of patients with poor functional outcomes was significantly higher in the higher-TSPO group (compared to the lower group) in terms of clinical worsening (odds ratio (OR) = 11.69, 95% confidence interval (CI) = 2.08–65.6), poor functional outcomes (OR = 10.5, 95% CI = 1.14–96.57), and severe disability (OR = 4.8, 95% CI = 1.20–19.13). Plasma TSPO may be intimately linked with disease progression and worse functional outcomes in acute ischemic stroke patients.

## 1. Introduction

Translocator protein 18 kDa (TSPO), formerly known as a peripheral benzodiazepine receptor, is now recognized as a receptor present throughout the body and brain [1]. Under normal physiological conditions, TSPO levels in the brain are very low and restricted to glial cells, astrocytes, and microglia. TSPO expression was reported to coincide with the process of microglial activation, which is associated with brain injury and neuroinflammatory conditions such as trauma, neurodegenerative diseases, and neuroinflammatory diseases [2, 3].

Recent studies on TSPO focused on detecting inflammatory cells in the brain by imaging techniques. By labeling PK-11195, a high-affinity and selective ligand of TSPO, with various radioisotopes (^3^H, ^11^C, ^123^I, and ^125^I), the TSPO distribution can be visualized and measured by imaging techniques, such as positron emission tomography (PET) and single-photon emission computerized tomography (SPECT) [4]. TSPO has been used as a biomarker of brain injury and inflammation in various neurological diseases, including dementia [5, 6], degenerative diseases [7–9], multiple sclerosis [10, 11], encephalitis [12, 13], and stroke [14–17].

Relationships between plasma TSPO levels and central nervous system (CNS) diseases have not been fully assessed in the literature so far. The aim of this study was to measure the level of TSPO in a small number of patients with acute ischemic stroke and to determine whether plasma TSPO levels reflect the degree of stroke severity and can predict stroke outcomes. Study results can be used as a basis of larger studies to further clarify this relationship.

## 2. Materials and Methods

### 2.1. Patients

Patients with moderate to severe acute ischemic stroke admitted to a stroke unit for intensive care were recruited. Cerebral infarction was defined as a sudden onset of a focal neurological deficit that persists beyond 24 h. All patients received brain computed tomography (CT) or magnetic resonance imaging (MRI) to document the presence of an infarction and the absence of hemorrhage. Stroke diagnoses were made by a neurologist, who examined the patient and reviewed the pertinent diagnostic tests, but was blinded to the results of the TSPO test. In total, 40 patients were recruited from July 2014 to July 2015. Two patients were excluded due to an active infection prior to the incident stroke.

This study was approved by the Institutional Review Board (IRB) of Shin Kong Wu Ho-Su Memorial Hospital (IRB number: 20140401R). All patients or their family provided informed consent.

### 2.2. Data Collection

Demographic information and risk factors (age, sex, smoking habit, status of diagnosed hypertension, diabetes mellitus, atrial fibrillation, left ventricular hypertrophy, and hyperlipidemia) were examined using questionnaires with face-to-face interviews, medical records, and laboratory data. Stroke etiology was classified according to Trial of ORG 10172 in Acute Stroke Treatment (TOAST) criteria. Patients were evaluated by National Institutes of Health Stroke Scale (NIHSS) scores on admission, during hospitalization, and at discharge. Only patients with an NIHSS score of >5 on admission were included. Functional outcomes were assessed by the modified Rankin Scale (mRS) and Barthal Index (BI). The NIHSS, mRS, and BI were assessed by a trained certified nurse who was also blinded to the TSPO data.

### 2.3. Determination of TSPO

We collected baseline fasting blood samples for TSPO measurement within 24 h after the incident stroke and during hospitalization (on days 8–10). Levels of TSPO were assessed by an enzyme-linked immunosorbent assay (ELISA; catalog number SEJ628Hu, Cloud-Clone Corp., USA) in the serum of the patients. The detection range was 0.313–20 ng/ml. A level of <0.127 ng/ml (the minimum detectable dose of this kit) was assigned as zero. Low- and high-TSPO groups were divided based on the median value.

### 2.4. Study Outcomes

Clinical worsening was defined as a 2-point increase in the NIHSS score or death during hospitalization. A poor functional outcome was defined as moderate disability (mRS ≥ 4) or death (mRS = 6). Severe disability with total dependence was defined as a BI score of ≤30 or death.

### 2.5. Statistical Analysis

Demographic and vascular risk factors were compared by Chi-squared tests if they were categorical variables or by two-sample *t*-tests if continuous variables. To compare TSPO levels between groups, the Wilcoxon rank-sum test was performed. We calculated the odds ratio (OR) and associated 95% confidence interval (CI) by a binary logistic regression to estimate the risk of clinical worsening, poor functional outcomes, and severe disability among subjects with higher TSPO levels versus those with lower TSPO levels. We used a multivariate analysis of variance (MANOVA) to compare NIHSS scores at discharge between the two TSPO groups after adjusting for NIHSS scores at admission. A receiver operating characteristic curve (ROC) analysis and area under the ROC curve (AUC) were applied to express a measure of accuracy of TSPO for predicting patient outcomes. We also determined the cutoff value for optimally predicting a defined outcome status. The sensitivity and specificity were calculated using the proposed cutoff value. SAS 9.4 (SAS, Cary, NC, USA) was used for all statistical analyses. A two-tailed *p* value of <0.05 was considered significant.

## 3. Results

Baseline characteristics of all participants are shown in Table 1. Thirty-eight patients (19 men and 19 women) with a mean age of 69.6 (standard deviation (SD), 14.9) years were included. The mean NIHSS score on admission was 16.7 (SD, 7.9). Stroke etiology was classified as large artery atherothrombosis in 24 (63.1%), cardioembolism in 12 (31.6%), and other determined causes in 2 patients (5.3%) (both were cervical artery dissection). Thirteen patients were treated with intravenous tissue plasminogen activator (t-PA; 31.6%). Baseline characteristics between lower- and higher-TSPO groups showed no significant differences (Table 1).

The distribution of TSPO titers ranged from 0 (undetectable in seven patients) to 1.38 ng/ml with a median of 0.46 ng/ml and an interquartile range (IQR) of 0.57 ng/ml. The lower- and higher-TSPO groups were thus divided based on the median value.

**Figure 1:**
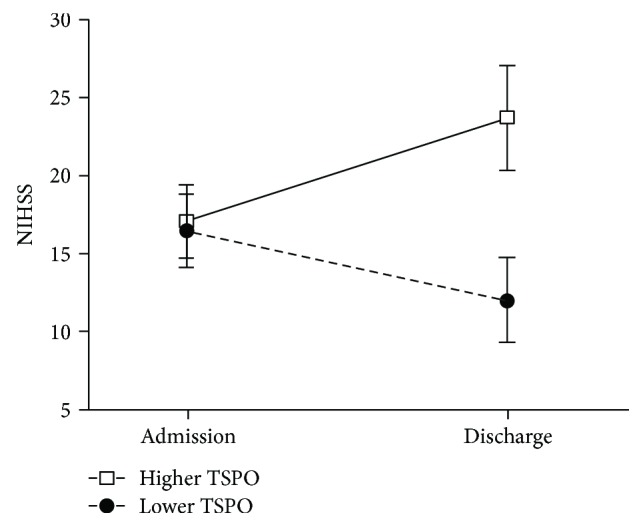
Changes of the mean National Institutes of Health Stroke Scale (NIHSS) scores at admission and discharge between the translocator protein (TSPO) groups. *p* = 0.0125 show the NIHSS scores of patients in the higher-TSPO group compared to those of the lower-TSPO group.

NIHSS scores at admission between the lower- and higher-TSPO groups showed no significant difference (mean NIHSS score 16.4 versus 17.1, *p* = 0.81), while at discharge, the NIHSS scores between the two TSPO groups had become significantly different (12.0 versus 23.7, *p* = 0.01) (Figure 1). An analysis by MANOVA after adjusting for the baseline NIHSS score showed a significant change between the two groups (*p* = 0.013).

**Figure 2:**
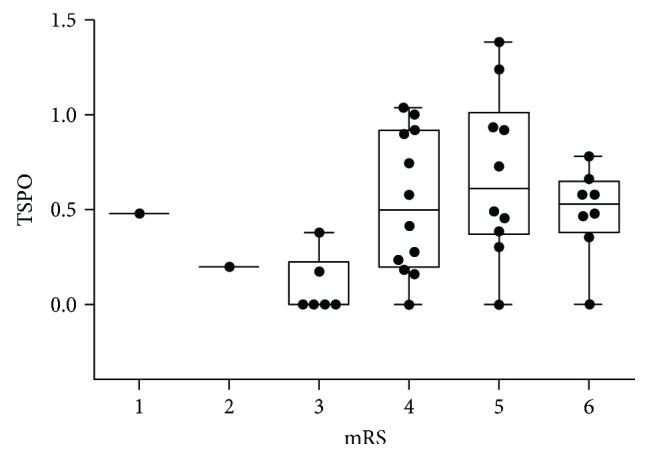
Boxplot of translocator protein (TSPO) titers by scores of the modified Rankin Scale (mRS).

In total, 13 patients (34.2%) had clinical worsening during hospitalization with a significantly higher proportion among those with higher TSPO (57.9% versus 10.5%, *p* = 0.001) and a strong risk (OR = 11.69, 95% CI: 2.08–65.6). mRS values at discharge were higher in the higher-TSPO group (4.79 versus 4.0, *p* = 0.02), while BI values were lower in the higher-TSPO group (19.2 versus 46.3, *p* = 0.02); both indicated worse outcomes. TSPO titers versus mRS scores are plotted in Figure 2 and show a clear difference between those with mRS scores of ≥4 and mRS scores of <4. The proportion of patients with poor functional outcomes as defined by mRS scores of ≥4 was significantly higher in the higher-TSPO group (94.7% versus 63.2%, *p* = 0.01, OR = 10.5, 95% CI, 1.14–96.57). Similarly, there were more patients with severe disability (BI scores of ≤30) in the higher-TSPO group (73.7% versus 36.8%, *p* = 0.02, OR = 4.8, 95% CI, 1.20–19.13). However, mortality rates between the lower- and higher-TSPO groups showed no significant difference (10.5% versus 31.5%, *p* = 0.23) (Table 2).

Further analysis with the ROC in Table 3 showed the good discriminative ability of the TSPO to classify clinical worsening, poor functional outcomes, and severe disability, with respective AUC values (*p* value) of 0.72 (*p* = 0.04), 0.84 (*p* = 0.001), and 0.70 (*p* = 0.03). The analysis also revealed that respective cutoff values of 0.46, 0.23, and 0.30 had the optimal prognostic accuracy with the best accuracy (sensitivity and specificity).

The clinical characteristics of patients with clinical worsening, poor functional outcomes, and severe disability are shown in Table 4. Initial NIHSS scores were correlated with severe disability. A history of hypertension, diabetes mellitus, and elevated creatinine and C-reactive protein (CRP) were correlated with clinical worsening. Diabetes was also correlated with poor functional outcomes. A lower hemoglobin level and elevated CRP were correlated with a more severe disability. However, only treatment with t-PA and TSPO titers were consistently correlated with all the three outcome measures. Median TSPO values were higher for clinical worsening (0.58 versus 0.30, *p* = 0.02), poor functional outcome (0.53 versus 0.08, *p* = 0.003), and severe disability (0.58 versus 0.19, *p* = 0.03).

There were 31 patients with second TSPO values sampled during hospitalization (on days 8–10). Values ranged from 0 (undetectable in 1 patient) to 1.38 ng/ml, with a median of 0.35 ng/ml and an interquartile range (IQR) of 0.40 ng/ml. Further analysis revealed the follow-up TSPO values were not correlated with any predefined outcome measures. The median (IQR) TSPO values on days 8-10 among stroke patients in without versus with clinical worsening (0.26 (0.38) versus 0.45 (0.34), median test, *p* = 0.11), in mRS of <4 versus mRS of ≥4 (0.25 (0.24) versus 0.48 (0.46), *p* = 0.20), in BI of >30 versus BI of ≤30 (0.24 (0.23) versus 0.40 (0.45), *p* = 0.12), and survival versus death (0.33 (0.39) versus 0.50 (0.22), *p* = 0.13) individually.

## 4. Discussion

### 4.1. Evidence of Elevated TSPO Levels in Stroke Patients

This is the first study to demonstrate that the TSPO level can be detected in the plasma of patients with acute ischemic stroke. The level of TSPO within 24 h after an incident stroke was associated with the appearance of clinical worsening and poor functional outcomes at discharge. It was accompanied by a good discriminative ability to detect clinical worsening, and poor mRS and BI scores, but not discharge mortality. However, the initial TSPO level was not correlated with the baseline stroke severity, suggesting that TSPO is not directly linked to the stroke location or the infarct size. Rather, it is the ongoing ischemic cascade and subsequent neuroinflammation that were related to outcomes in our patients.

### 4.2. TSPO Expression in Ischemic Stroke

Following ischemic injury to the brain, a series of neuroinflammatory responses ensues, which include the activation of endogenous CNS cells (astrocytes, neurons, and microglia) and an influx of leukocytes [18, 19]. TSPO expression is increased in microglia and in circulating macrophages entering the brain. Using a ligand specific for TSPO with an imaging study like SPECT or PET, regional increases in TSPO levels can be demonstrated within the infarct core and boundary zones [20, 21]. Early studies in ischemic animal models using 3H-PK 11195 autoradiography demonstrated that changes reached a maximum of 4–8 days after induction of local ischemia [22]. In a rat model of middle cerebral artery infarction, an increase in 3H-PK 11195 binding was found in the peri-infarct zone 7 days after insult, which was colocalized with increased glucose metabolism and accumulation of microglia and macrophages. This peri-infarct neuroinflammation might contribute to an extension of tissue damage [23]. There are only a limited number of clinical studies using TSPO ligand imaging. Gerhard et al. showed that areas of increased 11C-PK 11195 uptake were related to areas where T1-weighted MRI showed intensity changes, but over time, increased tracer binding involved the area of the primary lesion and areas distant from the infarct lesion [15, 24]. Price et al. [17] analyzed temporal and spatial patterns of microglial activation in four ischemic stroke patients, and showed that binding was identified beyond 72 h and extended to 30 days in the core infarction, contralateral hemisphere, and peri-infarct zone. Increased tracer binding was detected in the thalamus ipsilateral to the stroke in seven patients with chronic middle cerebral artery infarcts. Areas with intense binding were observed around subcortical lesions and along subcortical white matter tracts [16]. Those findings suggest that remote microglial activation in the weeks and months after a stroke might indicate Wallerian degeneration.

In summary, the extent of microglial activation is dependent on the time and severity of the insult, and increased TSPO expression seems to be associated with progression of tissue damage and increased risks of further injury. Local microglial activity in the area of the infarct was negatively correlated with clinical outcomes [25, 26].

### 4.3. Plasma Biomarkers and Ischemic Stroke

Unlike the use of diagnostic serum biomarkers, such as creatinine kinase and troponin, to evaluate acute coronary syndrome, the current diagnosis of stroke relies solely on a neurological examination and neuroimaging study. Serum biomarkers for the differential diagnosis, assessment of stroke severity, and predicating the prognosis are increasingly needed. Considerable interest has focused on brain-specific glial proteins (e.g., S100 calcium-binding protein B (S100B) and glial fibrillary acidic protein (GFAP)) and neuronal cells (e.g., ubiquitin C-terminal hydrolase- (UCH-) L1, neuron-specific enolase (NSE), *α*II-spectrin breakdown products- (SBDP-) 120, SBDP145, and SBDP150, myelin basic protein (MBP), neurofilament light chain (NF-L), tau protein, visinin-like protein-1 (VLP-1), and the NR2 peptide) that can be detected in the peripheral blood and provide valuable diagnostic information [27, 28]. For example, the concentration of S100B increases in the plasma of patients with ischemic stroke, and levels are associated with a larger infarct size, poor neurovascular status on admission, and worse outcomes [29]. On the other hand, plasma GFAP has been used to differentiate between intracranial hemorrhage (ICH) and ischemic stroke, with high sensitivity and specificity [30]. For neuron-specific biomarkers of acute brain injury, NSE has been most extensively investigated. Serum NSE levels at 72 h after a stroke were significantly correlated with worse neurological outcomes, and levels have high predictive value for determining stroke severity [31–33]. The diagnostic value of serum tau protein in ischemic stroke is controversial. While one study pointed that tau was correlated with the severity of neurological deficits and the infarct volume [34], another study concluded that early tau protein concentrations were not correlated with the degree of neurological deficits or disability in the acute stage or after 3 months [35].

The above clinical trials demonstrate that brain-specific proteins can be recovered from peripheral blood after brain injury and may reflect the degree of tissue injury in the brain. In contrast to the abovementioned brain-specific proteins, TSPO does not reflect the extent of brain injury but rather expresses subsequent inflammation, which makes it unique in predicting disease progression and worse outcomes. Although many of these biomarkers demonstrate independent diagnostic and prognostic values in ischemic stroke and ICH, the heterogeneity of stroke phenotypes suggests that useful clinical information can be obtained only from a panel of selected biomarkers. TSPO may be regarded as a promising biomarker that should be added to the current panel.

### 4.4. Limitations of this Study

The results of the present study are only preliminary. The main limitation of this study is its small sample size. The cutoff value for predicting outcome was susceptible to bias. Further, despite excluding patients with active infection at stroke onset, other factors that might contribute to elevated TSPO were not elucidated. We included only patients with moderate to severe stroke. TSPO expression in milder stroke patients is thus unavailable. Moreover, due to the IRB ethical board regulations, DNA evaluations of study samples were not feasible in this study. Studies on rs6971 polymorphism, in terms of drug ligand and cholesterol binding, seems to be necessary. Further studies with larger numbers of patients with different stroke subtypes and rs6971 polymorphism analysis are needed.

## 5. Conclusions

Plasma TSPO might reflect CNS inflammation after a stroke and may be intimately linked with disease progression and final outcomes, including clinical worsening and poor functional outcomes.

## Figures and Tables

**Table 1:** Baseline characteristics of 38 patients stratified into lower- and higher-translocator protein (TSPO) groups.

	Total	Lower TSPO	Higher TSPO	*p*
		(≤0.46 ng/ml)	(>0.46 ng/ml)	
*N*	38	19	19	
Female (%)	19	8 (42.1)	11 (57.9)	0.33
Age (years)	69.6 (14.9)	68.5 (13.4)	70.6 (16.5)	0.66
Glasgow Coma Scale	12.0 (3.8)	12.2 (3.7)	11.7 (4.1)	0.74
NIHSS score at the baseline	16.7 (7.9)	16.4 (8.7)	17.1 (7.4)	0.81
Hypertension (%)	27 (71.0)	12 (36.1)	15 (78.9)	0.28
Diabetes mellitus (%)	12 (31.5)	5 (26.3)	7 (36.8)	0.48
CAD (%)	9 (23.7)	3 (15.79)	6 (31.6)	0.25
CKD (%)	7 (18.4)	2 (10.5)	5 (26.3)	0.20
Smoker (%)	9 (23.7)	6 (31.58)	3 (15.8)	0.25
Hemoglobin (g/dl)	13.9 (2.7)	13.7 (3.2)	14.0 (2.1)	0.68
Platelets (10^3^/*μ*l)	215 (70)	209 (90)	221 (43)	0.61
WBCs (10^3^/mm^3^)	11.0 (4.8)	11.6 (5.5)	10.3 (4.0)	0.45
BUN (mg/dl)	22.1 (12.7)	21.1 (11.3)	23.3 (14.7)	0.68
Creatinine (mg/dl)	1.50 (1.8)	1.54 (2.19)	1.46 (1.16)	0.89
CRP (mg/dl)	5.07 (7.2)	3.23 (5.16)	6.91 (5.65)	0.28
TOAST classification				
Large artery atherothrombosis	24 (63.1)	12 (63.1)	12 (63.1)	1.0
Cardioembolism	12 (31.6)	6 (31.6)	6 (31.6)	
Other determined causes	2 (5.3)	1 (5.3)	1 (5.3)	
IV-t-PA (*n*, %)	13(31.6)	8(42.1)	5(26.3)	0.49

Values are the mean (SD) or *n* (%), as appropriate. NIHSS, National Institutes of Health Stroke Scale; CAD, coronary artery disease; CKD, chronic kidney disease; WBCs, white blood count; CRP, highly sensitive C-reactive protein; TOAST, Trial of ORG 10172 in Acute Stroke Treatment; IV-t-PA, intravenous tissue plasminogen activator.

**Table 2:** Clinical worsening and other outcome measures at discharge in relation to translocator protein (TSPO) levels.

	Total	Lower TSPO	Higher TSPO	*p*	OR (95% CI)
		(≤0.46 ng/ml)	(>0.46 ng/ml)		
Clinical worsening, *n* (%)	13 (34.2)	2 (10.5)	11 (57.9)	**0.001**	11.69 (2.08–65.6)
mRS, mean (SD)	4.39 (1.22)	4.00 (1.1)	4.79 (1.2)	**0.02**	
mRS ≥ 4, *n* (%)	30 (78.95)	12 (63.2)	18 (94.7)	**0.01**	10.50 (1.14–96.57)
BI, mean (SD)	32.8 (35.1)	46.3 (36.1)	19.2 (28.9)	**0.02**	
BI ≤ 30, *n* (%)	21 (55.2)	7 (36.8)	14 (73.7)	**0.02**	4.80 (1.20–19.13)
Death, *n* (%)	8 (20.05)	2 (10.5)	6 (31.5)	0.23	3.92 (0.67–22.7)

mRS, modified Rankin Scale; BI, Barthal Index; OR, odds ratio; CI, confidence interval; SD, standard deviation.

**Table 3:** Area under the receiver operating characteristic curve (AUC), cutoff, sensitivity, and specificity of the translocator protein (TSPO) in classifying prognostic outcomes of stroke patients.

	Cutoff	AUC	*p*	Sensitivity	Specificity
Clinical worsening	0.46	0.72	**0.04**	0.84	0.68
Poor functional outcome, mRS ≥ 4	0.23	0.84	**0.001**	0.83	0.75
Severe disability, BI ≤ 30	0.30	0.70	**0.03**	0.85	0.59

mRS, modified Rankin Scale; BI, Barthal Index.

**Table 4:** Clinical characteristics of patients with clinical worsening, poor functional outcomes, and severe disability.

	Clinical worsening			Poor function outcomes, mRS ≥ 4			Severe disability, BI ≤ 30		
	Yes	No	*p*	Yes	No	*p*	Yes	No	*p*
*N*	13	25		30	8		21	17	
Female (%)	8 (61.5)	11 (44.0)	0.30	16 (53.3)	3 (37.5)	0.69	13 (61.9)	6 (35.3)	0.10
Age (years)	71.6 (17.7)	68.4 (13.4)	0.53	71.1 (14.5)	63.2 (15.8)	0.90	70.8 (16.1)	67.8 (13.5)	0.54
NIHSS	16.9 (8.7)	16.6 (7.4)	0.91	17.7 (7.9)	12.8 (7.3)	0.11	19.0 (7.2)	13.9 (8.0)	**0.04**
HTN (%)	12 (92.3)	15 (60.0)	**0.03**	23 (76.7)	4 (50.0)	0.19	17 (80.9)	10 (58.8)	0.13
DM (%)	7 (53.8)	5 (20.0)	**0.03**	12 (40.0)	0 (0.0)	**0.03**	9 (42.8)	3 (17.6)	0.09
CAD (%)	4 (30.7)	5 (32.0)	0.69	7 (23.3)	2 (25.0)	1.0	5 (23.8)	4 (23.5)	0.98
CKD (%)	5 (38.4)	2 (8.0)	0.20	6 (20.0)	1 (12.5)	1.0	5 (23.8)	2 (11.7)	0.33
Smoker (%)	1 (7.69)	8 (32.0)	0.12	7 (23.3)	2 (25.0)	1.0	5 (23.8)	4 (23.5)	0.98
Hemoglobin (g/dl)	12.7 (3.17)	14.5 (2.3)	0.05	13.7 (3.0)	14.2 (1.3)	0.66	13.0 (3.2)	14.9 (1.5)	**0.02**
Platelets (10^3^/*μ*l)	212 (108)	215 (45)	0.98	219 (77)	197 (31)	0.45	218 (92)	209 (30)	0.69
WBC (10^3^/mm^3^)	12.0 (6.6)	10.5 (3.7)	0.37	10.2 (3.2)	11.3 (5.3)	0.60	11.6 (5.6)	10.0 (2.9)	0.37
BUN (mg/dl)	23.2 (14.7)	21.1 (11.2)	0.68	27.0 (19.0)	19.5 (7.2)	0.15	26.2 (3.4)	18.0 (3.4)	0.09
Creatinine (mg/dl)	2.41 (2.85)	1.05 (0.41)	**0.02**	1.66 (1.96)	0.94 (0.17)	0.31	1.92 (2.3)	1.03 (0.36)	0.12
CRP (mg/dl)	10.9 (10.1)	2.8 (4.3)	**0.02**	6.0 (7.9)	1.7 (1.1)	0.30	8.48 (9.0)	1.66 (1.0)	**0.03**
IV-t-PA (%)	1 (7.69)	12 (48.0)	**0.01**	8 (26.7)	5 (62.5)	**0.09**	1 (4.7)	12 (70.6)	**0.001**
TSPO	0.58 (0.53)	0.30 (0.57)	**0.02**	0.53 (0.61)	0.08 (0.33)	**0.003**	0.58 (0.47)	0.19 (0.61)	**0.03**

Values are mean (standard deviation) or *n* (%), as appropriate. TSPO values (ng/ml) are median (interquartile range). NIHSS, National Institutes of Health Stroke Scale; HTN, hypertension; DM, diabetes mellitus; CAD, coronary artery disease; CKD, chronic kidney disease; WBC, white blood count; CRP, highly sensitive C-reactive protein; IV-t-PA, intravenous tissue plasminogen activator; TSPO, translocator protein.

## Data Availability

The datasets used and analyzed in the current study are available from the corresponding author upon request.
